# Alterations in white matter volume and integrity in obesity and type 2 diabetes

**DOI:** 10.1007/s11011-016-9792-3

**Published:** 2016-01-27

**Authors:** Liselotte van Bloemendaal, Richard G. Ijzerman, Jennifer S. ten Kulve, Frederik Barkhof, Michaela Diamant, Dick J. Veltman, Eelco van Duinkerken

**Affiliations:** Diabetes Center / Department of Internal Medicine, VU University Medical Center, PO BOX 7057, 1007 MB Amsterdam, The Netherlands; Department of Radiology & Nuclear Medicine, VU University Medical Center, 1007 MB Amsterdam, The Netherlands; Department of Psychiatry, VU University Medical Center, 1007 MB Amsterdam, The Netherlands; Department of Medical Psychology, VU University Medical Center, 1007 MB Amsterdam, The Netherlands; Department of Psychology, Pontifícia Universidade Católica (PUC-Rio), Rio de Janeiro, RJ Brazil

**Keywords:** Brain, White matter, Volume, Integrity, Type 2 diabetes, Obesity, DTI, VBM

## Abstract

Type 2 diabetes mellitus (T2DM) is characterized by obesity, hyperglycemia and insulin resistance. Both T2DM and obesity are associated with cerebral complications, including an increased risk of cognitive impairment and dementia, however the underlying mechanisms are largely unknown. In the current study, we aimed to determine the relative contributions of obesity and the presence of T2DM to altered white matter structure. We used diffusion tensor imaging (DTI) and voxel-based morphometry (VBM) to measure white matter integrity and volume in obese T2DM patients without micro- or macrovascular complications, age- gender- and BMI-matched normoglycemic obese subjects and age- and gender-matched normoglycemic lean subjects. We found that obese T2DM patients compared with lean subjects had lower axial diffusivity (in the right corticospinal tract, right inferior fronto-occipital tract, right superior longitudinal fasciculus and right forceps major) and reduced white matter volume (in the right inferior parietal lobe and the left external capsule region). In normoglycemic obese compared with lean subjects axial diffusivity as well as white matter volume tended to be reduced, whereas there were no significant differences between normoglycemic obese subjects and T2DM patients. Decreased white matter integrity and volume were univariately related to higher age, being male, higher BMI, HbA1C and fasting glucose and insulin levels. However, multivariate analyses demonstrated that only BMI was independently related to white matter integrity, and age, gender and BMI to white matter volume loss. Our data indicate that obese T2DM patients have reduced white matter integrity and volume, but that this is largely explained by BMI, rather than T2DM *per se*.

## Introduction

Obesity and type 2 diabetes mellitus (T2DM) are major public health problems, not only due to their pandemic occurrence, but also due to their association with adverse consequences, such as cardiovascular disease, cancer and cerebral complications (Kullmann et al. 2015; Geijselaers et al. 2015). T2DM is characterized by hyperglycemia and obesity-related insulin resistance (Kahn 2003; Kahn et al. 2006). Patients with T2DM are at an increased risk of (vascular) dementia (Crane et al. 2013), stroke (Aoki and Uchino 2011), white matter lesions (Roriz-Filho et al. 2009) and cognitive impairment (Benedict et al. 2012; Reijmer et al. 2010). Furthermore, T2DM is related to loss of grey and white matter volume (Moran et al. 2013) and to loss of white matter integrity (Reijmer et al. 2010). Although less well established, obesity is also associated with brain disease, including an increased risk of dementia and accelerated cognitive decline at older age, with complementary structural brain changes (Gustafson et al. 2003; Gunstad et al. 2007; Kullmann et al. 2015). Both obesity-related insulin resistance and hyperglycemia seem strong risk factors for cerebral pathology (Rusinek and Convit 2014).

Studies assessing brain alterations in T2DM usually include a heterogeneous sample of patients in early and more advanced stages of the disease, i.e., with clinically manifest microvascular and cardiovascular complications. It is therefore not yet clear to what extent structural brain changes are present in early stages of T2DM. Imaging studies in obese subjects have likewise included heterogeneous or inadequately characterized samples with regard to glucose tolerance, insulin resistance, and sometimes cardiovascular disease (Rusinek and Convit 2014).

In the current study, we aimed to determine the relative contributions of obesity and the presence of non-complicated T2DM to altered white matter volume and integrity. We therefore performed voxel-based morphometry (VBM) and diffusion tensor imaging (DTI) in obese T2DM patients, BMI-matched normoglycemic obese subjects and normoglycemic lean subjects. We hypothesized that white matter integrity and volume are altered in obese T2DM patients compared with BMI-matched normoglycemic obese subjects and lean subjects.

## Methods

### Participants

This study, part of a larger study (NCT01281228), was approved by the Medical Ethics Committee of the VU University Medical Center and was performed in accordance with the Helsinki Declaration. All participants provided written informed consent before participation. We included 16 obese T2DM patients, 16 obese normoglycemic and 16 healthy lean individuals. Inclusion and exclusion criteria have been reported previously (van Bloemendaal et al. 2014). Briefly, inclusion criteria included right-handedness, BMI >30 kg/m^2^ for obese individuals and T2DM patients, BMI <25 kg/m^2^ for lean controls, normoglycemia for obese individuals and lean controls as defined by fasting plasma glucose <5.6 mmol/l and 2-h glucose <7.8 mmol/l following a 75 g oral glucose tolerance test (OGTT). For T2DM patients, HbA1c had to be 6.0–8.5 %. Exclusion criteria included cardiovascular diseases, micro-albuminuria, neurological or psychiatric disorders including depression (assessed by Center for Epidemiologic Studies Depression scale) (Schroevers et al. 2000), substance abuse or use of any centrally acting agent. Microalbuminuria was tested by calculating the albumin: creatinine ratio in urine, and was defined as an albumin:creatinine ratio >2.5 for men or >3.5 for women.

### Data acquisition

MRI scanning was performed on a 3.0 Tesla GE Signa HDxt scanner (General Electric, Milwaukee, Wisconsin, USA) using an 8-channel phased-array head coil. For this study we used an echo planar imaging based DTI acquisition consisting of 5 volumes without directional weighting and 30 volumes with 30 non-collinear diffusion gradient directions (b-value 1000 s/mm^2^, repetition time (TR) 6200 ms, echo time (TE) 81 ms, 45 contiguous axial slices of 2.4 mm). Participants furthermore had a T2-based Fluid Attenuating Inverse Recovery (3D-FLAIR; TR 8000 ms, TE 126 ms, slice thickness 1.2 mm) and a T1-weighted fast spoiled gradient-echo (TR 8.2 ms, TE 3.2 ms, 1 mm slice thickness) sequence.

### DTI tract-based analysis

Processing of DTI-scans was performed using the FMRIB’s Diffusion Toolbox of FMRI’s Software Library (FSL) version 5.04 (http://fsl.fmrib.ox.ac.uk/fsl/fslwiki) (Smith et al. 2004). First, DTI scans and gradient-vectors were corrected for motion and eddy-current induced distortions (Leemans and Jones 2009). Next, the diffusion tensor was calculated for each voxel, providing fractional anisotropy (FA), axial (λ1), radial (mean of λ2 and λ3) and mean diffusivity (mean of λ1, λ2 and λ3) (Pierpaoli and Basser 1996; Basser and Jones 2002). Tract-based spatial statistics (TBSS) was used for voxelwise statistical analysis, which does not use spatial smoothing (Smith et al. 2006). All individual FA images were non-linearly registered to FMRIB58FA standard space, to allow group averaging and comparison. These registered images were then averaged and the mean image was skeletonized, and thresholded at 0.2 to include only white matter. Individual non-linear warps and skeleton projection of FA-images were used to project axial, radial and mean diffusivity to the skeleton and to allow voxelwise statistics. All steps were manually checked, no errors occurred.

### VBM analysis

T1-weighted images were visually inspected for motion artefacts. Data preprocessing was performed with Statistical Parametric Mapping 8 software (SPM8; Wellcome Trust Center for Neuroimaging, London, UK; http://www.fil.ion.ucl.ac.uk/spm). First, structural images were oriented along the anterior/posterior commissure axis and segmented into grey matter, white matter and cerebrospinal fluid with a bias-field correction cut-off of 60 mm full width at half maximum (FWHM). In the next step, DARTEL (Diffeomorphic Anatomical Registration Through Exponentiated Lie Algebra) was used to create a group-specific grey and white matter template and the individual flow fields. These flow fields contain the non-linear deformations between each individual’s MRI-scan and the DARTEL template. The individual grey and white matter segmentations were registered to MNI standard space using linear affine registration and non-linear deformation using the flow fields. The images were modulated to preserve relative volume and corrected for brain size, in order to allow group comparisons. Lastly, the segmented, modulated and normalized images were smoothed using an 8 mm FWHM Gaussian kernel. Both segmentation and normalization results were manually checked, no errors occurred.

### White matter lesions

White matter lesions were scored visually by an experienced neuro-radiologist (FB) based on 3D-FLAIR sequence using Fazekas criteria (Fazekas et al. 1987).

### Statistical analyses

Clinical group data are expressed as mean ± SEM (unless otherwise stated) and were analyzed with the Statistical Package for the Social Sciences (SPSS) version 20 (SPSS, Chicago, IL, USA). Between group differences in clinical data, including white matter lesions, were analyzed using a one-way ANOVA with Bonferroni post-hoc correction or *χ*^2^-test whether appropriate.

Differences in DTI parameters between groups were analyzed with the FSL function “randomise”, using threshold-free cluster enhancement non-parametric permutation testing (5000 permutations). This method does not require a minimum cluster size to be defined a priori. Family Wise Error (FWE) was applied as multiple comparisons correction. For the VBM analysis of white matter volume, clusters were considered relevant if at least 150 voxels at P_uncorrected_ < 0.002. Those clusters were subsequently whole brain cluster-wise corrected for multiple comparisons using FWE. This minimum of 150 voxels ensured that only larger, more relevant alteration patterns were found, reducing the risk of false-positive findings and increasing the reliability of the results.

Both the DTI and VBM analyses were corrected for age, gender and systolic blood pressure. A *P*-value <0.05 after FWE-correction was considered statistically significant.

Using FSL the mean value of white matter integrity of voxels differing between groups was extracted. For each subject the mean white matter volume of significant clusters was extracted using MarsBar 0.41 (http://marsbar.sourceforge.net). Using Spearman’s ρ the demographical and clinical correlates of altered volume/integrity were determined. These correlations were calculated in all participants. To determine the strongest predictors, variables significantly correlating with volume/integrity, were entered in a forward regression model.

## Results

### Group demographics

Due to technical problems, DTI scans were not available for 1 lean control and 1 obese subject. All subjects were matched for age and gender, while T2DM patients and normoglycemic obese subjects were also BMI matched (Table 1). Patients with T2DM had higher systolic blood pressure, HbA1c, fasting plasma glucose and insulin levels compared with normoglycemic obese and lean subjects. Total cholesterol and LDL cholesterol levels were lower in T2DM patients compared with obese and lean subjects, whereas HDL cholesterol levels were higher in lean compared with T2DM and obese subjects. Eight T2DM patients were treated with metformin monotherapy and 8 used metformin in combination with a sulfonylurea. Twelve T2DM patients and 3 obese subjects used antihypertensive medication, 13 T2DM patients and 1 obese subject used statins. There were no significant differences in Fazekas score (white matter lesions) between the groups (*P* = 0.8) (Table 1).

**Table 1:** Subject characteristics

	Lean (*n* = 15)	Obese (*n* = 15)	T2DM (*n* = 16)	ANOVA *P*-value
Age (years)	57.3 ± 1.9	57.7 ± 2.2	61.4 ± 1.5	0.2
Gender, male/female (n)	8/7	8/7	8/8	–
Weight (kg)	71.5 ± 2.9	100.7 ± 3.0*	97.9 ± 3.0*	<0.001
Body mass index (kg/m^2^)	23.4 ± 0.4	32.6 ± 0.8*	34.0 ± 0.9*	<0.001
Waist circumference (cm)	85.7 ± 2.1	112.3 ± 2.2*	115.7 ± 1.8*	<0.001
Systolic blood pressure (mmHg)	119 ± 4	127 ± 3	141 ± 3†	<0.001
Diastolic blood pressure (mmHg)	76 ± 2	79 ± 2	83 ± 2	0.057
Fasting plasma glucose (mmol/l)	5.2 ± 0.1	5.3 ± 0.1	8.4 ± 0.5†	<0.001
Glucose 2 h after OGTT (mmol/l)	5.1 ± 0.3	5.4 ± 0.2	–	0.4
HbA1c (%)	5.5 ± 0.03	5.5 ± 0.06	6.9 ± 0.22†	<0.001
HbA1c (mmol/mol)	37 ± 0.3	37 ± 0.7	52 ± 2.4†	<0.001
Total cholesterol (mmol/l)	5.6 ± 0.2	5.6 ± 0.2	4.5 ± 0.3†	0.002
LDL-cholesterol (mmol/l)	3.3 ± 0.2	3.4 ± 0.2	2.3 ± 0.2†	<0.001
HDL-cholesterol (mmol/l)	1.9 ± 0.1	1.4 ± 0.1*	1.3 ± 0.1*	<0.001
Triglycerids (mmol/l)	0.9 ± 0.1	1.7 ± 0.3	1.8 ± 0.3	0.046
Fasting NEFA (mmol/l)	0.46 ± 0.04	0.45 ± 0.03	0.64 ± 0.04†	0.001
Fasting insulin (pmol/l)	36 ± 2.8	81 ± 14	117 ± 17†	<0.001
Diabetes duration (years)	–	–	7.0 [4.25, 10.75]	–
Fazekas score (0; 1; 2; 3)	6; 7; 2; 0	8; 5; 2; 0	5; 9; 2; 0	0.8

Data are means ± SEM or median [interquartile range]

Fazekas score for white matter lesions: 0 indicates no lesions; 1 indicates punctate foci; 2 indicates beginning confluence of foci; 3 indicates large confluent areas

*OGTT* oral glucose tolerance test, *NEFA* non-esterified fatty acids, *T2DM* type 2 diabetes patients

*Statistically significant different from lean (post-hoc Bonferroni corrected *P* < 0.05)

†Statistically significant different from lean and obese (post-hoc Bonferroni corrected *P* < 0.05)

### White matter integrity

**Fig. 1:**
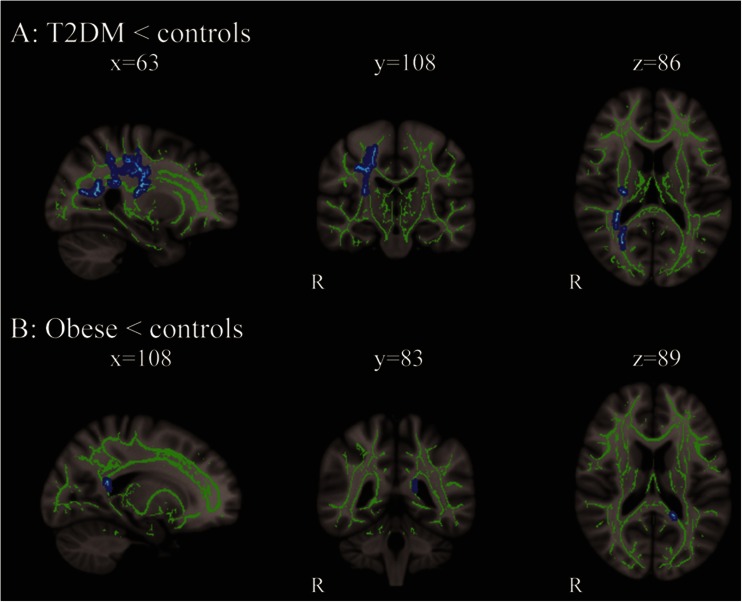
Changes in axial diffusivity in T2DM patients and obese compared with lean subjects. **a** Brain slices showing decreased axial diffusivity (λ_1_) in T2DM patients compared with lean subjects in right corticospinal tract, right inferior fronto-occipital tract, right superior longitudinal fasciculus and right forceps major; **b** Brain slices showing a cluster of voxels on the left side of the forceps major where axial diffusivity tended to be lower in subjects with obesity compared with controls (P_FWE_ = 0.11). The mean skeleton is shown in green, and significant differences are displayed as thickened tracts in blue for visualization purposes. Left side of the axial slices is the right side of the brain. X, y, z are the Montreal Neurological Institute (MNI) coordinates of the brain in standard space

DTI tract-based analyses showed significantly lower axial diffusivity (λ_1_) in T2DM patients compared with lean subjects. Tracts most affected were the right corticospinal tract, right inferior fronto-occipital tract, right superior longitudinal fasciculus and right forceps major (Fig. 1a). On the left side of the forceps major there was a cluster of voxels where axial diffusivity tended to be lower in subjects with obesity compared with lean controls (P_FWE_ = 0.1; Fig. 1b). There were no significant differences in FA, mean diffusivity and radial diffusivity between T2DM patients, normoglycemic obese and lean subjects.

### White matter volume

**Fig. 2:**
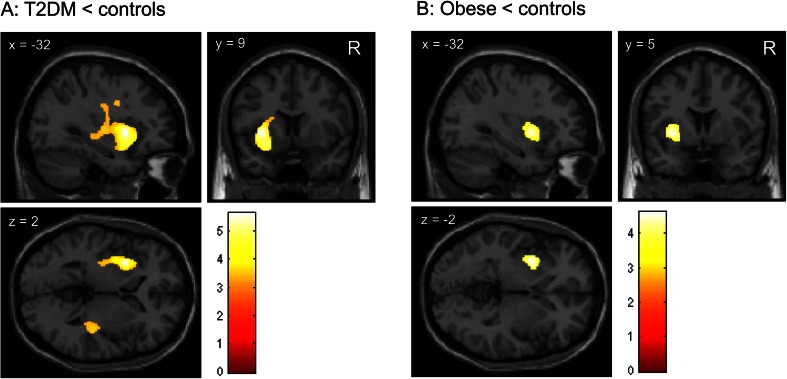
Reduced white matter volume in T2DM patients compared with lean subjects. **a** Brain slices showing clusters of reduced white matter volume in the external capsule region in obese T2DM patients compared with lean subjects, as determined with VBM; **b** Brain slices showing a cluster of reduced white matter volume in the external capsule region in obese compared with lean subjects. This cluster, however, was not statistically significant after FWE-correction for multiple comparisons (P_FWE_ = 0.380). The color scale reflects the T-value. Right side of the axial slices is the right side of the brain. X, y, z are the Montreal Neurological Institute (MNI) coordinates of the brain in standard space

VBM analyses demonstrated significantly lower white matter volume in T2DM patients relative to lean control subjects in a left hemisphere cluster comprising predominantly the external capsule region. Another cluster of lower white matter volume in T2DM patients versus lean control subjects was found in the right inferior parietal lobe (Fig. 2a; Table 2).

**Table 2:** Between-group differences in white matter volume and integrity

	Region	Side	Cluster (voxels)	Volume (mL)	T	P-_uncorr_	P-_FWE_	MNI coordinates (x, y, z)
White matter volume								
T2DM < Lean	External capsule	L	5735	19.36	5.64	<0.001	<0.001	−32,9,2
					4.99	<0.001	<0.001	−27,8,−13
					4.03	<0.001	<0.001	17,−16,33
	Inferior Parietal Lobe	R	3276	11.06	4.69	<0.001	<0.001	45,−42,54
					4.49	<0.001	<0.001	47,−31,30
					4.48	<0.001	<0.001	39,−12,36
Obese < Lean	External capsule	L	903	3.05	4.62	<0.001	0.380	−32,5,−1
Axial diffusivity								
T2DM < Lean	Corticospinal tract, inferior fronto-occipital tract, superior longitudinal fasciculus and forceps major	R	1803	1.80	5.49	NA	<0.05	22,−28,46

*T* t-statistic, *P-*_*FWE*_ *p*-value Family-Wise Error corrected for multiple comparisons, *R* right, *L* left, *MNI* Montreal Neurological Institute coordinates in mm

*NA* not applicable

In normoglycemic obese compared with lean subjects, lower white matter volume was found in the left external capsule region as well (Fig. 2b; Table 2), although this cluster was smaller than the cluster found in T2DM patients. In addition, this cluster was not statistically significant after FWE-correction for multiple comparisons (P_FWE_ = 0.380). No differences were identified in the comparison between normoglycemic obese subjects and T2DM patients.

### Associations between white matter parameters and biomedical variables

For each subject the mean axial diffusivity value was extracted for the significant voxels in the comparison between T2DM patients and lean controls. The same was done for white matter volume of the two VBM clusters that reached statistical significance when comparing T2DM patients with lean control subjects. With these values correlations with demographic and clinical characteristics were calculated in the overall group. As can be found in Table 3, univariate correlations of lower axial diffusivity with higher BMI, fasting plasma glucose, fasting insulin levels and HbA1c were significant. Of these variables, higher BMI was the only independent predictor of lower axial diffusivity (β = −0.48; *P* = 0.001), which explained 21 % of the variance. Lower white matter volume in the left external capsule cluster was univariately related to higher age, BMI, fasting plasma glucose and insulin levels, HbA1c, and being male. Being male (β = 0.432; *P* < 0.001), higher BMI (β = −0.353; *P* = 0.002) and higher age (β = −0.342; *P* = 0.003) were independently related with lower white matter volume in this cluster. This model explained 47 % of the variance of white matter volume differences in this cluster. Similar univariate correlations were found for the cluster of lower white matter volume in the right inferior parietal lobe, with the exception of fasting plasma glucose (Table 2). Independent predictors were gender (being male; β = 0.449; *P* < 0.001), higher age (β = −0.345; *P* = 0.004), and higher BMI (β = −0.284; *P* = 0.016), together explaining 44 % of the variance.

**Table 3:** Associations between white matter parameters and demographic and clinical characteristics

	Axial diffusivity^a^		White matter volume cluster L^b^		White matter volume cluster R^b^	
	Spearman’s rho	*P*-value	Spearman’s rho	*P*-value	Spearman’s rho	*P*-value
Age	−0.09	0.573	−0.34	0.021	−0.30	0.041
Gender	0.27	0.069	0.52	<0.001	0.54	<0.001
BMI	−0.47	0.001	−0.55	<0.001	−0.43	0.003
Fasting glucose	−0.45	0.002	−0.31	0.035	−0.24	0.10
Fasting insulin	−0.44	0.002	−0.40	0.006	−0.30	0.040
HbA1c	−0.38	0.008	−0.42	0.004	−0.38	0.010
HDL	0.27	0.07	0.17	0.3	0.15	0.35

^a^Mean axial diffusivity value for the significant voxels in the comparison between T2DM patients and lean controls

^b^Mean white matter volume of the two significant VBM clusters in the comparison between T2DM patients and lean controls. With these values correlations with biomedical variables were calculated in all subjects (lean, obese and T2DM)

## Discussion

In the current study we showed that both white matter integrity, as measured by axial diffusivity, and white matter volume are decreased in obese T2DM patients compared with lean subjects. In normoglycemic obese compared with lean subjects axial diffusivity as well as white matter volume tended to be reduced, whereas there were no significant differences between normoglycemic obese subjects and T2DM patients. Higher BMI independently predicted decreased white matter integrity, and, together with higher age and being male, it predicted lower white matter volume as well. Higher HbA1C, fasting plasma glucose and insulin levels were no independent predictors of decreased white matter volume and integrity.

Using DTI, we found that obese T2DM patients compared with lean subjects have lower axial diffusivity in the right corticospinal tract, right inferior fronto-occipital tract, right superior longitudinal fasciculus and right forceps major. Our findings are in line with previous studies showing lower axial diffusivity in type 1 diabetes patients with and without microvascular complications in comparable tracts (van Duinkerken et al. 2012) and in T2DM patients (Hoogenboom et al. 2014), although the latter study only observed a trendwise reduced axial diffusivity in the cingulum bundle. In the current study, we combined DTI with VBM of white matter and found that T2DM patients have reduced white matter volume in the right inferior parietal lobe and the left external capsule region. A previous study in a large cohort of T2DM patients (of which 24 % had known cardiovascular and 11 % cerebrovascular complications), also demonstrated white matter loss in T2DM, but mainly in frontal and temporal regions (Moran et al. 2013). In addition, in obese adolescents with T2DM versus BMI-matched non-diabetic subjects, reduced frontal and whole brain white matter volume has been demonstrated, paralleled by reductions in cognitive performance (Yau et al. 2010).

To determine the relative contributions of obesity and T2DM to structural brain changes, we also studied normoglycemic obese subjects (BMI-matched with the T2DM patients). We found in normoglycemic obese compared with lean subjects reduced axial diffusivity, as well as reduced white matter volume, but these differences in white matter integrity and volume were only trendwise significant after strict FWE-correction for multiple comparisons. It could be speculated that obesity is associated with early white matter alterations and that hyperglycemia may further impact brain white matter structure. However, in the current study we found no significant differences between obese T2DM patients and obese normoglycemic subjects in white matter structure.

Relating our findings of localized lower white matter volume and integrity to specific functions is not straightforward given the complexity of brain networks. However, it is well known that the corticospinal tracts have a major role in motor coordination, whereas the forceps major and parietal cortices have been related to the transfer and processing of somatosensory information. In a previous study in T2DM patients white matter integrity of the inferior longitudinal fasciculus has been related to cognitive functioning, mainly information processing speed (Reijmer et al. 2013). Similar correlations between the inferior fronto-occipital tract and cognition were found in type 1 diabetes patients (van Duinkerken et al. 2012). Our finding of reduced white matter volume in the external capsule region in obese T2DM patients furthermore corroborates previous findings of white matter alterations in the external capsule in obese versus lean women (Shott et al. 2015). The external capsule connects medial and ventral prefrontal cortices with limbic regions, contains fibers from both the inferior fronto-occipital fasciculus and uncinate fasciculus, and connects the hippocampus and amygdala with prefrontal and OFC regions (Shott et al. 2015; Schmahmann et al. 2008). Future studies are needed to better understand the consequences of the observed changes in white matter volume and integrity in our study.

We did not observe any differences in FA or any of the other diffusion tensor parameters. Previous studies have shown alterations in FA or mean and radial diffusivity in T2DM (Hoogenboom et al. 2014; Hsu et al. 2010; Reijmer et al. 2013). In the current study we only observed reduced axial diffusivity in T2DM patients. The biological substrate of axial diffusivity has mainly been derived from animal studies, and therefore careful interpretation is required. These animal studies, however, suggest that changes in axial diffusivity may represent changes in integrity of axons (Madden et al. 2009; Wheeler-Kingshott and Cercignani 2009). This could result from a less favorable alignment of fibers within the bundle, but it could also represent damage to the axons themselves. In a mouse model of multiple sclerosis it was demonstrated that greater decreases in axial diffusivity were associated with greater amounts of axonal damage and with more neurological disability (Budde et al. 2008). Further studies are needed to determine the clinical relevance of loss of axial diffusivity in T2DM patients.

Results from the white matter volume analysis appear to be more robust and extensive than those of the white matter integrity analysis. This may seem counterintuitive as white matter integrity is considered to be more sensitive to early alterations than white matter volume. The difference, in this case, may be due to our statistical approach. For the white matter volume analysis, first clusters with a *P* < 0.002 and a minimum size of 150 voxels were identified. Those clusters were subsequently whole brain cluster-wise corrected for multiple comparisons using FWE. For the white matter integrity analysis this approach is not available and a whole-brain voxel-wise FWE-correction was used. Obviously, the latter approach is more stringent and will yield less statistically significant voxels. We chose the cluster-wise approach for the volumetric analysis given the small sample size. These different approaches need to be taken into account when comparing both results.

In the overall group we found univariate correlations between altered white matter integrity and volume, age, being male, BMI, and fasting glucose and insulin levels. These correlations suggest that hyperglycemia and insulin-resistance partly relate to obesity/T2DM related cerebral alterations. However, in a multivariate model only BMI was related to white matter integrity, and age, gender and BMI to white matter volume loss. The association between higher BMI and lower white matter volume is in line with a previous study in elderly subjects (Raji et al. 2010). Obesity is characterized by a chronic proinflammatory state (Johnson et al. 2012) and low-grade inflammatory markers have been shown to be related to white matter integrity and other brain parameters (Verstynen et al. 2013; Frodl and Amico 2014). In this study no peripheral or central proinflammatory markers were available, but future research should investigate this issue.

We also determined correlations between white matter integrity/volume and HDL and LDL cholesterol. In the overall group, the mean value of the cluster of lower axial diffusivity, but not white matter volume, tended to correlate positively with both cholesterol types. While the association with HDL cholesterol is as anticipated, the direction of the correlation with LDL seems counterintuitive (higher LDL correlates with higher axial diffusivity values). This finding may however be explained by the statin use in the obese and T2DM group, which lowers LDL cholesterol levels. Therefore, this correlation is likely to be unreliable in our current study design. Ideally, such a correlation should be calculated in individuals not using statins, which was not feasible in the current study due to power issues. We have therefore excluded LDL from the analyses.

For exploratory purposes we have added, besides BMI, other biomedical variables, such as fasting insulin and glucose, to a second level statistical design to test whether regional white matter integrity/volume is related to these variables. However, given the study design, focusing on groups differing with regard to T2DM and BMI, these tests will not be fully independent of BMI. This is a different approach than the approach we took by correlating clusters of altered integrity/volume to biomedical variables as a post-hoc analysis. This second level whole brain statistical analysis did not show any statistically significant correlations between white matter integrity/volume and fasting plasma glucose or fasting plasma insulin.

A strength of this study is that groups were well-phenotyped as we only included patients with non-complicated T2DM, BMI-matched normoglycemic subjects and healthy lean subjects. A limitation of our study is the relatively small sample size, limiting the power to detect significant differences between the groups. However, despite the small sample size we observed significant differences between T2DM patients and lean subjects in measures of white matter tract integrity and white matter volume. Another limitation of the current study is that measurements of cognitive functions were not performed. Previous studies have demonstrated associations between reduced white matter volume/integrity and cognitive functions in T2DM as well as in obesity (Hoogenboom et al. 2014; Moran et al. 2013; Reijmer et al. 2013; Zhang et al. 2014; Kullmann et al. 2015). Reduced white matter integrity and volume may play a key role in obesity- and T2DM-related cognitive impairment (Kullmann et al. 2015).

In conclusion, we found that both white matter integrity and white matter volume are focally decreased in obese patients with non-complicated T2DM compared with lean subjects. In normoglycemic obese compared with lean subjects axial diffusivity as well as white matter volume only tended to be reduced. Higher BMI was an independent predictor of decreased white matter integrity as well as white matter volume. Our data indicate that obese T2DM patients have reduced white matter integrity and volume, but that this is largely explained by BMI, rather than the presence of T2DM *per se*.
